# Choriocapillaris and Choroidal Microvasculature Imaging with Ultrahigh Speed OCT Angiography

**DOI:** 10.1371/journal.pone.0081499

**Published:** 2013-12-11

**Authors:** WooJhon Choi, Kathrin J. Mohler, Benjamin Potsaid, Chen D. Lu, Jonathan J. Liu, Vijaysekhar Jayaraman, Alex E. Cable, Jay S. Duker, Robert Huber, James G. Fujimoto

**Affiliations:** 1 Department of Electrical Engineering and Computer Science, and Research Laboratory of Electronics, Massachusetts Institute of Technology, Cambridge, Massachusetts, United States of America; 2 Lehrstuhl für BioMolekulare Optik, Ludwig-Maximilians-Universität München, Munich, Germany; 3 Advanced Imaging Group, Thorlabs, Inc., Newton, New Jersey, United States of America; 4 Praevium Research Inc., Santa Barbara, California, United States of America; 5 New England Eye Center and Tufts Medical Center, Tufts University, Boston, Massachusetts, United States of America; Medical University Graz, Austria

## Abstract

We demonstrate *in vivo* choriocapillaris and choroidal microvasculature imaging in normal human subjects using optical coherence tomography (OCT). An ultrahigh speed swept source OCT prototype at 1060 nm wavelengths with a 400 kHz A-scan rate is developed for three-dimensional ultrahigh speed imaging of the posterior eye. OCT angiography is used to image three-dimensional vascular structure without the need for exogenous fluorophores by detecting erythrocyte motion contrast between OCT intensity cross-sectional images acquired rapidly and repeatedly from the same location on the retina. *En face* OCT angiograms of the choriocapillaris and choroidal vasculature are visualized by acquiring cross-sectional OCT angiograms volumetrically via raster scanning and segmenting the three-dimensional angiographic data at multiple depths below the retinal pigment epithelium (RPE). Fine microvasculature of the choriocapillaris, as well as tightly packed networks of feeding arterioles and draining venules, can be visualized at different *en face* depths. Panoramic ultra-wide field stitched OCT angiograms of the choriocapillaris spanning ∼32 mm on the retina show distinct vascular structures at different fundus locations. Isolated smaller fields at the central fovea and ∼6 mm nasal to the fovea at the depths of the choriocapillaris and Sattler's layer show vasculature structures consistent with established architectural morphology from histological and electron micrograph corrosion casting studies. Choriocapillaris imaging was performed in eight healthy volunteers with OCT angiograms successfully acquired from all subjects. These results demonstrate the feasibility of ultrahigh speed OCT for *in vivo* dye-free choriocapillaris and choroidal vasculature imaging, in addition to conventional structural imaging.

## Introduction

While retinal vasculature supplies oxygen and nutrients to the inner retinal layers, choroidal vasculature is responsible for nourishing the outer retinal layers. Choroidal circulation, the largest source of the blood supply to the posterior eye, is also responsible for transporting metabolic waste from the retinal pigment epithelium (RPE), and hence it plays a key role in normal retinal function [1]. Choroidal circulation is known to be associated with retinal diseases such as age-related macular degeneration (AMD) and diabetic retinopathy (DR) [2], [3], which are major causes of vision loss or impairment. Therefore, visualization of the microvasculature and blood flow in the choriocapillaris, the capillary layer of the choroid, is of great interest.

Imaging the choriocapillaris *in vivo* is challenging because of highly pigmented choroidal melanocytes and its location below the RPE. Furthermore, unlike retinal blood vessels, capillaries in the choriocapillaris are fenestrated [4], which makes high resolution choriocapillaris imaging with fluorescein and indocyanine green (ICG) angiography more challenging due to dye leakage. As a result, most studies of the choriocapillaris have been limited to postmortem histology or indirect observations such as dye perfusion pattern or time measurement.

Multiple histological studies have investigated the interrelation between photoreceptors, RPE, Bruch's membrane, and choriocapillaris in AMD. By analyzing donor eyes with geographic atrophy (GA), Sarks *et al.* found that RPE loss is associated with atrophy of the choriocapillaris and choroid [5]. A smaller study by McLeod *et al.* suggested a linear relationship between the loss of RPE and choriocapillaris in non-exudative AMD patients with GA [6]. A later study by Mullins *et al.* reported that choriocapillaris density decreases in association with sub-RPE deposit formation [7]. These and other histological studies suggest that choriocapillaris atrophy is associated with RPE atrophy. Although alterations in the choriocapillaris are expected to be secondary to changes in the RPE in non-exudative AMD according to histological studies [5], [8], it is still possible that one of the earliest changes detectable in the retina *in vivo* could be alterations in choriocapillaris circulation and/or structure. Furthermore, a number of studies report that the location of drusen formation is associated with the underlying choriocapillaris and choroidal vessel structure, thus suggesting that drusen are more likely to form where choroidal perfusion is insufficient [2], [9]–[11]. In addition, in exudative AMD, changes in the choriocapillaris may occur before changes in the RPE, and could be the primary cause of sub-retinal neovascularization [6]. A number of histological and electron micrograph corrosion casting studies also show choriocapillaris degeneration associated with diabetes [3], [12]–[15]. Therefore, the ability to image choriocapillaris and larger choroidal vessels *in vivo* may be useful for the better understanding of pathophysiology, early diagnosis, treatment response monitoring, and pharmaceutical development.

Optical coherence tomography (OCT) can provide depth-resolved information on the posterior eye and is gaining acceptance as a tool for *in vivo* choroidal imaging. However, the majority of studies on the choroid using OCT have focused on choroidal thickness measurements or imaging larger choroidal vessels, and to date, only a few studies have demonstrated choriocapillaris imaging consistent with histological and electron micrograph corrosion casts findings, in terms of capillary density. Using OCT phase information, Kurokawa *et al.* demonstrated choriocapillaris imaging in normal subjects with adaptive optics OCT near the fovea over a very limited field of view, due to limited imaging speed and constraints imposed by adaptive optics [16]. Using OCT phase variance and hardware eye tracking with a second scanning laser ophthalmoscope channel in an OCT system, Braaf *et al.* demonstrated OCT choriocapillaris angiography of the central fovea from a single normal subject, although the acquisition time per volume was relatively long compared to clinical standards, and multiple volumes were used for averaging [17]. In general, choriocapillaris imaging *in vivo* using OCT is a relatively unexplored area, and it requires extensive further investigation to relate it to known histological findings in normal and pathological eyes.

In the present study, we investigate *in vivo* choriocapillaris imaging using ultrahigh speed, speckle variance (decorrelation) OCT angiography. We image different fundus locations and depths to compare results with known histological and electron micrograph corrosion casting studies. OCT angiography enables the investigation of choriocapillaris vascular structure *in vivo*. Combined with conventional depth-resolved structural imaging capabilities of OCT, OCT angiography promises to be a powerful tool to visualize and investigate the outer retinal layers and choriocapillaris comprehensively in retinal diseases.

## Materials and Methods

### Study Population

The human study protocol was approved by the Committee on the Use of Humans as Experimental Subjects at the Massachusetts Institute of Technology (MIT) and was in accordance with the Declaration of Helsinki. Written informed consent forms were obtained from all subjects after explaining the study. Eight normal volunteers (mean age 33.0±11.1 years, range 24 to 54 years) without any known history of retinal diseases participated in the study. All imaging was performed at MIT.

### Ultrahigh Speed Swept Source OCT System

The ultrahigh speed swept source OCT (SSOCT) prototype used in this experiment is similar to one recently developed by our group, as described in reference [18]. Therefore, only key characteristics or differences in the system are described here. The prototype SSOCT instrument uses a vertical cavity surface emitting laser (VCSEL) swept light source, which generates 400,000 unidirectional wavelength sweeps per second, resulting in an ultrahigh speed OCT A-scan rate of 400 kHz. The wavelength tuning range was ∼80 nm centered at ∼1060 nm, which provides a 9.6 µm full width at half maximum (FWHM) axial resolution in tissue. OCT signals are acquired with an analog-to-digital acquisition card (Alazar Technologies, ATS9360), which is externally clocked at uniform wavenumber intervals using a Mach-Zehnder interferometer output. The maximum clock frequency generated by the interferometer is set at 1.2 GHz, resulting in a total imaging range of ∼2.44 mm in tissue, which is sufficient for posterior eye imaging. The incident optical power at the cornea is ∼1.85 mW and the system sensitivity measured with a calibrated neutral density filter is ∼98.2 dB. The measured 3 dB sensitivity roll-off (∼70% in linear scale) range is >2 mm. Low sensitivity roll-off combined with deeper light penetration at 1060 nm wavelength, when compared to standard 850 nm, is important for deep choroidal imaging. Although OCT phase information was not used in this study, the system was still phase-stabilized by using a fiber Bragg grating to remove undesired fixed pattern noise [18]. The incident beam size on the cornea was ∼1 mm FWHM, thus yielding an estimated ∼10 µm FWHM spot size on the retina, corresponding to a ∼24 µm Airy disk diameter. However, diffraction from aberrations in the eye is expected to yield a larger spot size.

### Data Acquisition

Ultrahigh speed SSOCT imaging was performed in one eye of each subject. Imaging was performed with room lights off, without pupil dilation. Two different fundus locations were imaged in all subjects, the central fovea and ∼6 mm temporal to the fovea. An internal fixation target could be used for imaging these two fundus locations. For one subject, multiple fundus locations spanning ±16 mm from the central fovea was imaged to obtain a panoramic montage visualization of the choriocapillaris layer. The internal fixation target could not be used for fundus locations outside of ±6.5 mm from the central fovea. In those cases, a small light emitting diode (LED) was used as an external fixation target. For each fundus location, two different retinal field sizes were imaged, 1.5 mm×1.5 mm and 3 mm×3 mm. For both field sizes, 4 B-scans were acquired from the same position repeatedly, from 400 B-scan positions in order to generate blood flow contrast, thereby resulting in a total of 1600 B-scans per data set. Each B-scan consisted of 800 A-scans. The time interval between sequential B-scans was ∼2.4 milliseconds. The total acquisition time was ∼3.8 seconds per volumetric data set. The 1.5 mm field size provides higher sampling density, and thus better image quality, while the 3 mm field provides larger coverage on the retina.

### Swept Source OCT Data Processing

Each OCT signal acquisition consisted of 928 samples, which yielded 464 pixels per A-scan in the axial direction after Fourier transformation. The sample spacing was ∼5 µm per pixel in depth, which was ∼2× smaller than the axial resolution of the system.

### Choriocapillaris OCT Angiography

**Figure 1:**
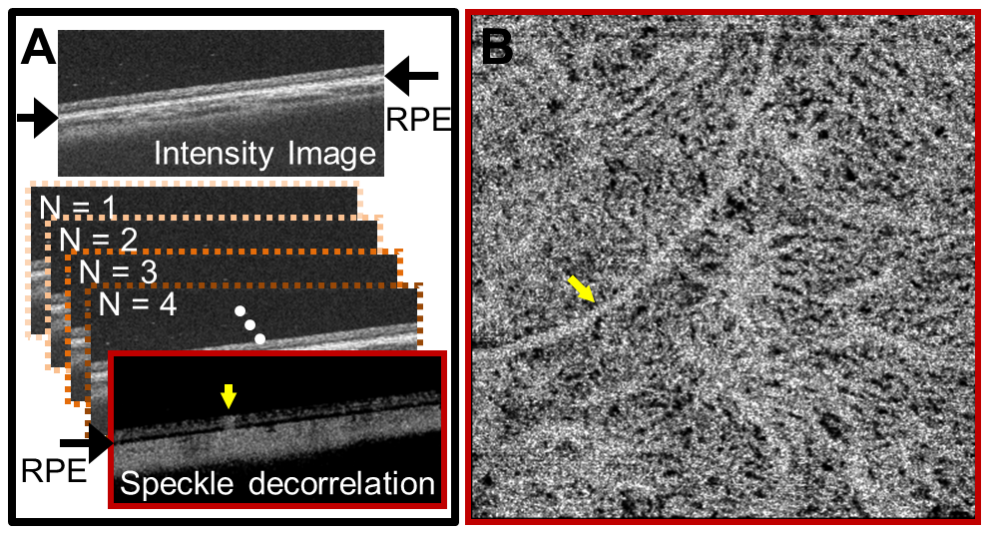
Generating OCT angiography data from OCT intensity data. (A) Multiple OCT intensity B-scans are acquired from the same location on the retina and compared with each other pixel-by-pixel to detect motion contrast. The resultant OCT cross-sectional angiogram is segmented with respect to the RPE using one of the corresponding intensity images. (B) By performing the steps in (A) volumetrically and displaying the OCT angiography data in an *en face* plane below the RPE, the choriocapillaris microvasculature can be visualized. Yellow arrows indicate shadowing artifact from thick retinal vessels.

OCT angiograms that display contrast from blood flow were generated by calculating cross-sectional images of the speckle decorrelation between the 4 B-scans repeatedly acquired from the same position with a 2.4 millisecond time interval. If the imaged tissue is locally stationary (in the axial and transverse directions), all 4 B-scans will be locally identical. However blood vessels will have erythrocyte motion. Because the 4 B-scans are acquired at slightly different times, this motion generates a locally fluctuating back-scattered intensity within the blood vessels in the B-scans. This fluctuation in back-scattered intensity is calculated by comparing the 4 B-scans on a pixel-by-pixel basis (Figure 1A). We did not correct for bulk-motion due to involuntary patient eye motion, assuming that the erythrocyte travel speeds are much faster than the patient bulk motion. However, it is possible to correct for the effects of axial bulk motion and to suppress spurious decorrelations from transverse motion, if necessary. By performing this operation for all transverse positions, a volumetric OCT angiographic data set can be obtained from the volumetric intensity data set. In this study, we used OCT intensity information to calculate motion contrast. Similar techniques for generating motion contrast images based on OCT intensity and/or phase information have been described by several research groups [19]–[27].

Because the choriocapillaris layer is extremely thin, it is necessary to visualize the three-dimensional angiographic data set in an *en face* plane in order to resolve choriocapillaris features. The RPE was segmented by using the OCT intensity cross-sectional images in the volumetric data set. Because the angiographic and intensity volumes are from the same data set and are co-registered, the same RPE location can be used to segment the angiographic volume. *En face* OCT angiograms of the choriocapillaris at different depths, with respect to the RPE, were generated from the OCT angiographic volume (Figure 1B).

## Results and Discussion

**Figure 2:**
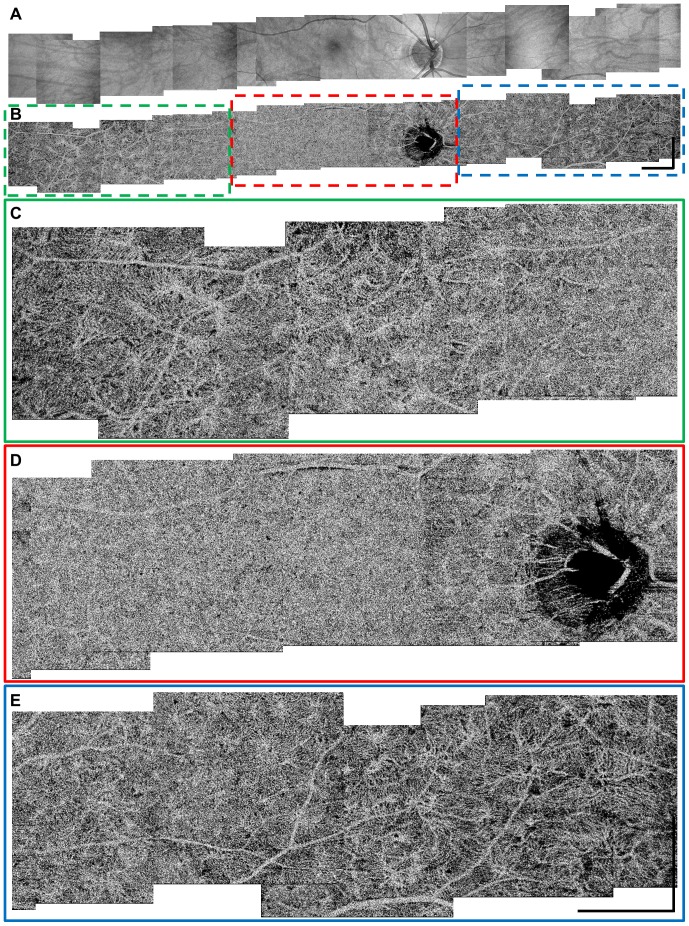
Panoramic wide field of view OCT angiogram of the choriocapillaris spanning ∼32 mm on the retina. (A) Stitched OCT intensity *en face* projection images. (B) Stitched OCT angiograms of the choriocapillaris. Different microvasculature patterns and densities can be observed at different fundus locations. (C–E) Close-up views of the OCT angiograms of the choriocapillaris in (B) for better visualization. Scale bars: 1.5 mm.

Figures 2A and 2B show a panoramic ultra-wide field of view (∼32 mm) OCT intensity projection image and an OCT angiogram of the choriocapillaris generated by stitching multiple smaller 3 mm×3 mm fields acquired from a healthy normal subject. The OCT *en face* angiograms were extracted from a depth immediately below the RPE. Previous histological and electron micrograph corrosion casting studies show that the capillary density and pattern in the choriocapillaris changes depending on the fundus location, with a densely packed honeycomb structure at the central fovea, and a more lobular and less dense structure towards the equator and periphery [13], [28]–[30]. The wide field *in vivo* choriocapillaris angiogram in Figure 2B demonstrates a spatial variation of the microvasculature consistent with these *ex vivo* postmortem studies. Figures 2C–E are close-up views of OCT angiograms taken from the different regions shown in Figure 2B that are used to visualize structural details better. Figure S1 shows the same images from Figures 2A and 2B with a higher pixel density.

As seen in the cross-sectional speckle decorrelation angiogram in Figure 1A and in the choriocapillaris *en face* angiogram in Figure 1B, large retinal vessels (marked with yellow arrows) tend to cast shadow-like artifacts in the axial direction. Since blood is highly scattering, large retinal blood vessels result in lower signal level below the vessels and more rapidly fluctuating speckle pattern below them, which cause the shadow-like artifacts in the angiographic data. Therefore, it is important to remember that large retinal vessels can produce shadow artifacts in choriocapillaris angiograms and are not part of choroidal vasculature.

Because the choriocapillaris has fine features in the *en face* plane, high A-scan sampling density and high speed are essential to visualize the microvasculature clearly. The current scan pattern of 800(horizontal)×400(vertical)×4(repeats) A-scans over 3 mm×3 mm corresponds to a 7.5 µm sample spacing in the vertical direction and 3.25 µm in the horizontal direction. Since the FWHM spot size on the retina is ∼10 µm, this corresponds to a moderate oversampling of ∼1.33× in the vertical direction and ∼2.66× in the horizontal direction, relative to the estimated spot size on the retina. The A-scan rate of the current prototype is 400 kHz. Slower speeds would result in lower image quality due to undersampling or would require trading off the field of view and acquisition time. This emphasizes the importance of imaging speed for *en face* imaging in general, and in particular motion contrast imaging, since it requires scanning the same location repeatedly.

**Figure 3:**
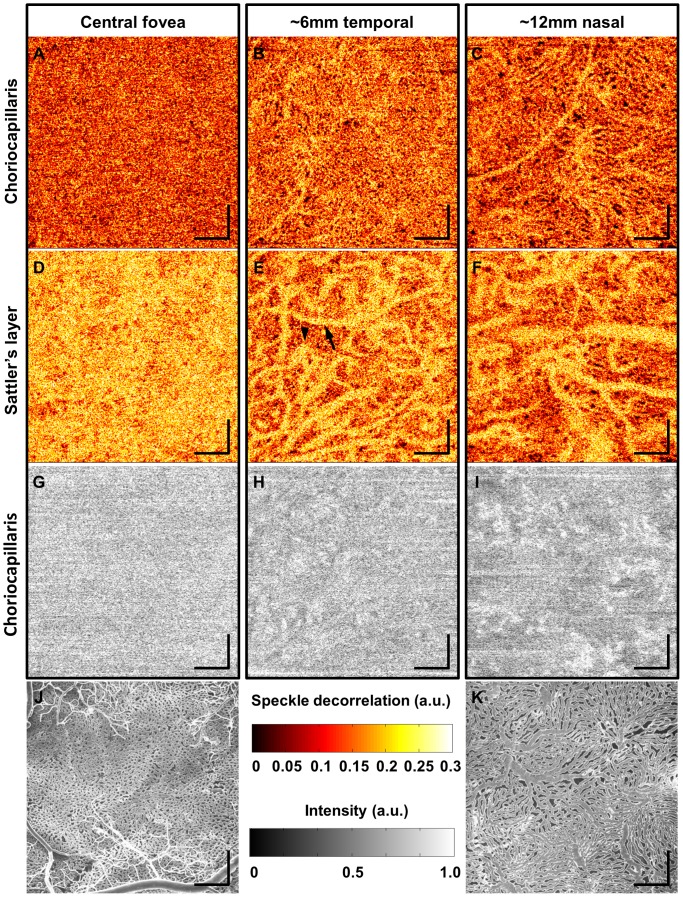
1.5×1.5 mm *en face* OCT angiograms at three different fundus locations. (A,D,G) OCT angiogram of the choriocapillaris, OCT angiogram of the Sattler's layer, and *en face* OCT intensity image (slice) corresponding to the choriocapillaris layer, respectively, at the central fovea. (B,E,H) OCT angiogram of the choriocapillaris, OCT angiogram of the Sattler's layer, and *en face* OCT intensity image (slice) corresponding to the choriocapillaris layer, respectively, at ∼6 mm temporal to the central fovea. The arrowhead indicates venules and arrow arteriole in (E). (C,F,I) OCT angiogram of the choriocapillaris, OCT angiogram of the Sattler's layer, and *en face* OCT intensity image (slice) corresponding to the choriocapillaris layer, respectively, at ∼12 mm nasal to the central fovea. (J,K) Electron micrographs of corrosion casts reproduced from Olver *et al.* with permission. Scale bars: 250 µm.

Figures 3A–C show 1.5 mm×1.5 mm *en face* choriocapillaris angiograms at three different fundus locations from the same normal subject in Figure 2. The smaller field size provides better image quality due to higher sampling density. The differences in capillary density and pattern between different fundus locations are clearly visualized. At the central fovea, the capillaries have a honeycomb structure with tightly packed vessel density (Figure 3A). At ∼6 mm temporal to the central fovea, a more lobular and less dense capillary structure can be identified (Figure 3B). At ∼12 mm nasal to the central fovea, the capillary structure becomes even less dense and with larger lobules (Figure 3C). Figures 3D–F are *en face* angiograms extracted from the same volumetric data sets but at ∼25–30 µm below the choriocapillaris, where feeding arterioles and draining venules in the Sattler's layer are clearly visualized. As in the choriocapillaris, the density of feeding and draining vessels tend to decrease and the vessel diameter increase at fundus locations away from the central fovea. Figures 3J and 3K, adapted from Olver *et al.* [29], show electron micrograph corrosion casts of the human choriocapillaris at the posterior pole and equator, respectively, which are displayed in the same scale as in the OCT angiograms for comparison. There is an excellent correspondence of structural features between the OCT angiograms in Figures 3A and 3C and the electron micrograph corrosion casts in Figures 3J and 3K, thus suggesting that OCT angiography enables *in vivo* visualization of the choriocapillaris.

Figure 3 shows false color images with varying degrees of speckle decorrelation. Although Figure 2 can also be displayed in false color, we chose to display only Figure 3 in false color to illustrate that it is particularly useful for identifying feeding arterioles and draining venules. Since speckle decorrelation imaging is based on motion contrast, the degree of speckle decorrelation increases if the erythrocyte speed is faster. Therefore, the feeding and draining vessels tend to have higher speckle decorrelation than capillaries, as seen in Figure 3. It is less clear how to differentiate feeding arterioles from draining venules when using only OCT angiography. However, previous morphology studies show that venules are centrally located and arterioles are located at the periphery of lobules [13], [28]. Using this information, some venules and arterioles can be identified from the lobular structure of the choriocapillaris, as shown in Figure 3E.

Figures 3G–I also show *en face* intensity images corresponding to the *en face* angiograms in Figures 3A–C. Because OCT intensity and angiography volumes are from the same data set, they are co-registered, and it is possible to extract *en face* angiograms and intensity images from precisely the same depth. As can be seen, the *en face* intensity images do not exhibit the choriocapillaris pattern seen in the OCT angiograms, which suggests that the intensity image alone cannot easily visualize the vessel lumen and stroma in the choriocapillaris. Intensity images have speckle noise that makes visualization of fine features difficult. However, OCT angiograms can visualize microvasculature in the choriocapillaris by displaying motion contrast from the fluctuation in intensity versus time. This points out that motion-contrast OCT angiography, whether it is using intensity or phase fluctuation information, is necessary to visualize the choriocapillaris structure. However, it should be noted that larger choroidal vessels below the choriocapillaris layer can still be visualized with *en face* OCT intensity images because their larger size and high flow rates generate contrast from light scattering and fringe washout effects. This is a different type of contrast mechanism from OCT angiography using speckle decorrelation. Video S1 demonstrates *en face* OCT angiography and intensity flythroughs in depth for comparison. The top and bottom rows show *en face* OCT angiograms and intensity images, respectively, at the central fovea, ∼6 mm temporal, and ∼12 mm nasal to fovea, from left to right.

Several previous studies have investigated the choriocapillaris using *en face* OCT intensity images. Motaghiannezam *et al.* visualize the choriocapillaris and larger choroidal vessels in the Sattler's and Haller's layers in three normal subjects using SSOCT at the 57 kHz A-scan rate [31]. Sohrab *et al.* also studied the choriocapillaris and larger choroidal vessel patterns with commercial SDOCT in patients with early AMD or reticular pseudo-drusen [32]. It should be noted that, while the methods and results in these studies could be clinically useful, OCT angiograms can be more sensitive than *en face* OCT intensity images for visualizing the choriocapillaris vascular structure. However, it should also be emphasized that the intensity images at greater depths showing larger choroidal vessels probably still reflect the vasculature, since they can be visualized with both OCT intensity and motion contrast angiography imaging.

**Figure 4:**
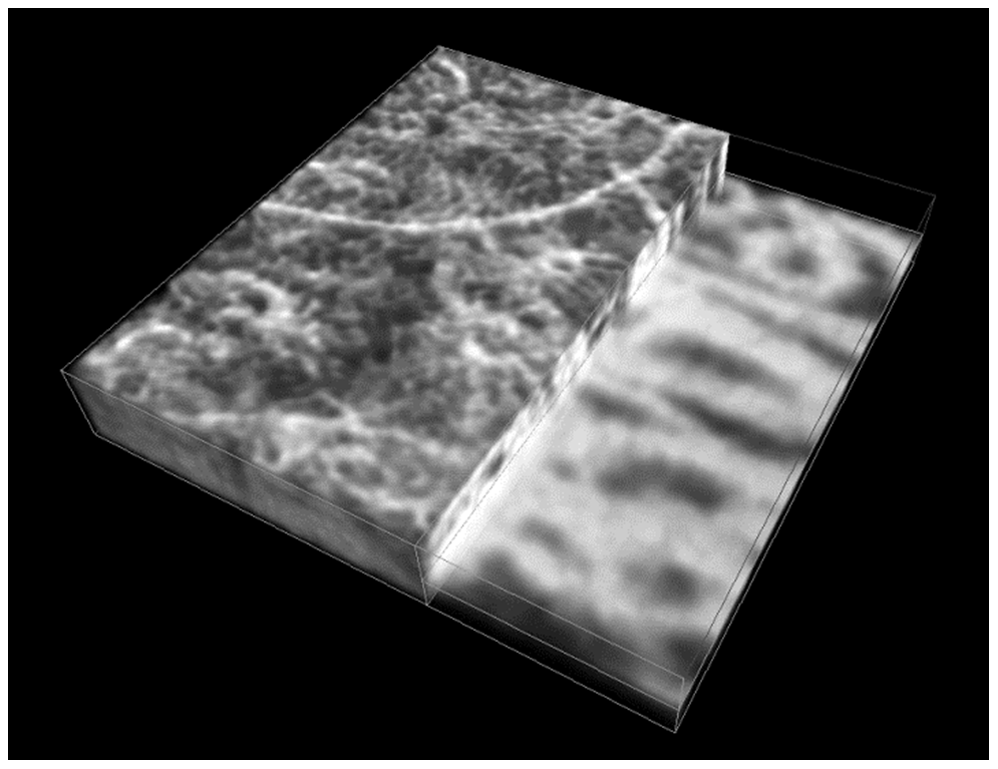
Three-dimensional rendering of a volumetric angiography data set covering a 1.5 mm×1.5 mm field on the retina. The locations where feeding arterioles and draining venules branch out to the choriocapillaris can be visualized more easily in three-dimensional rendering. One side of the rendering shows only depths below the choriocapillaris to emphasize the depth-resolving capability of OCT angiography.

Figure 4 is a three-dimensional rendering of volumetric OCT angiographic data acquired from a 1.5 mm×1.5 mm field of view at ∼12 mm nasal to the central fovea in order to emphasize that OCT angiography is indeed depth-resolved. One side of the volumetric angiogram rendering shows only structures below the choriocapillaris, while the other side shows the choriocapillaris and deeper structures. The rendering below the choriocapillaris layer shows the large feeding and draining vessels, while the rendering at the choriocapillaris layer shows the fine microvasculature in the choriocapillaris. The locations where the larger choroidal vessels in the Sattler's layer branch out to the choriocapillaris can also be identified in the rendering.

**Figure 5:**
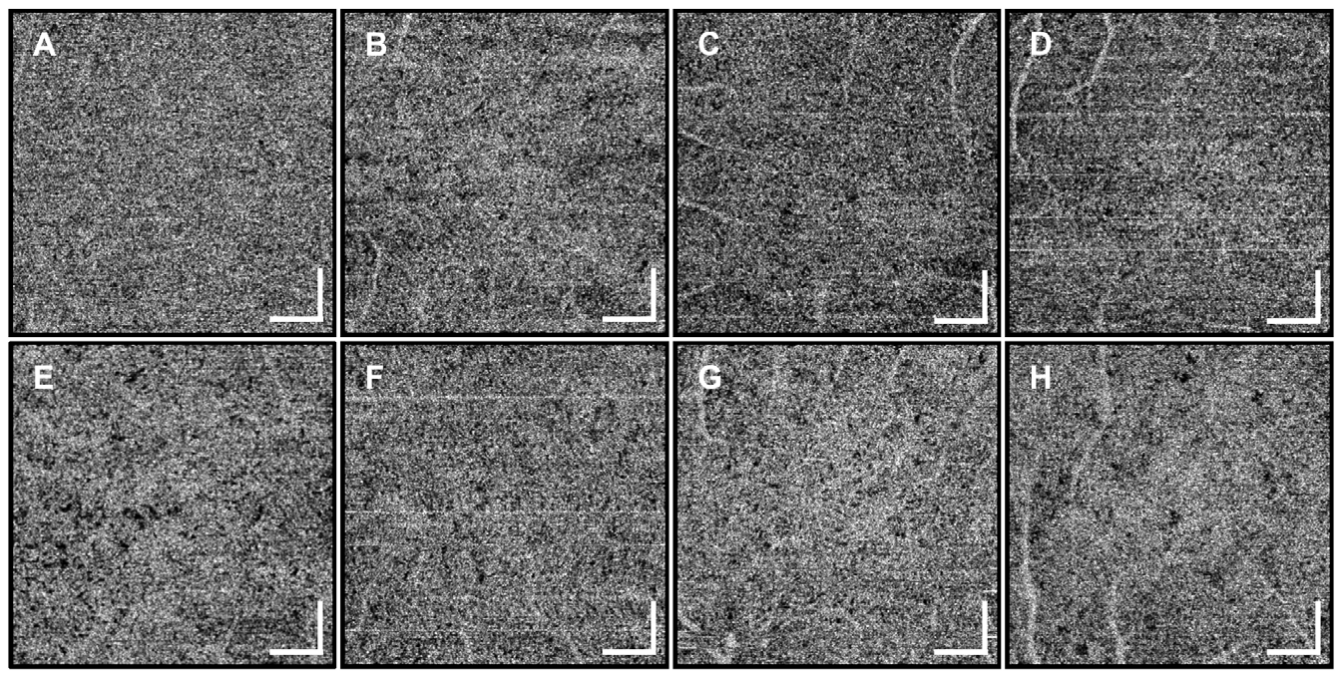
1.5×1.5 mm *en face* OCT angiograms at the central fovea for eight normal subjects. The choriocapillaris can be visualized in all subjects. Some variation between individuals can be noticed. The angiogram in (A) is the same image as in Figure 3(A) but in grayscale. Scale bars: 250 µm.

**Figure 6:**
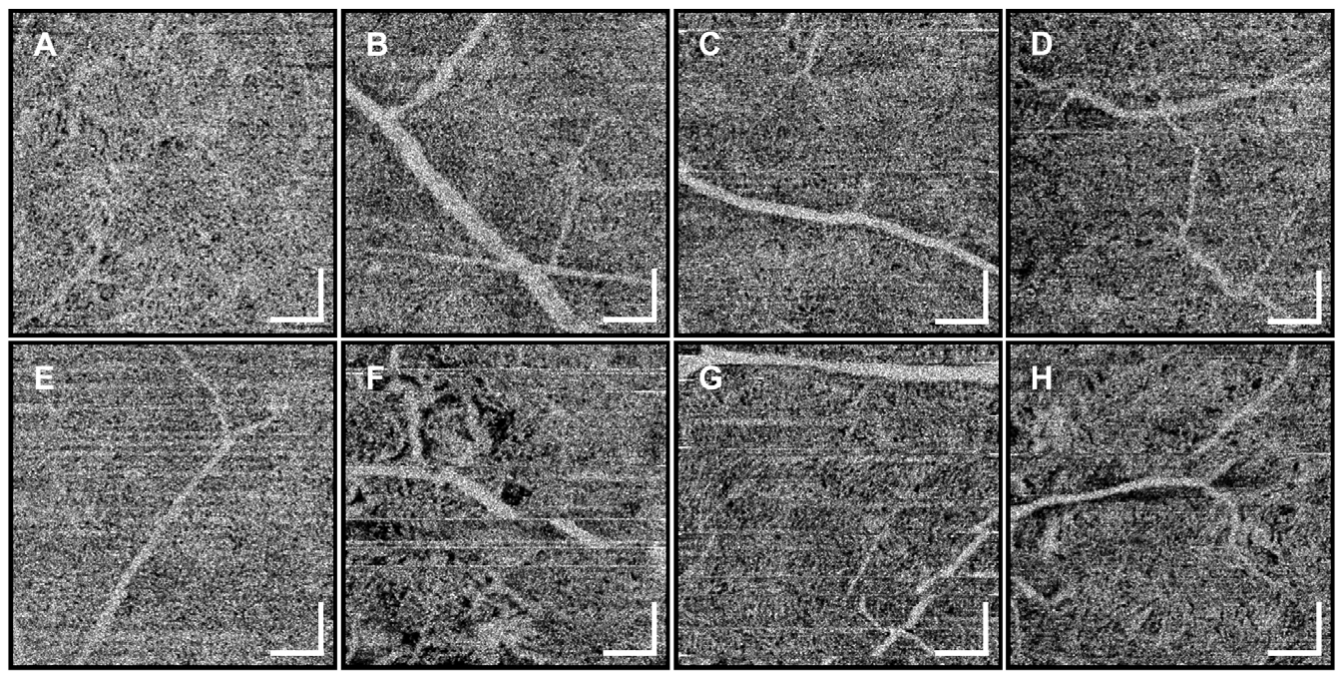
1.5×1.5 mm *en face* OCT angiograms at ∼6 mm temporal to the fovea for eight normal subjects. The choriocapillaris can be visualized in all subjects. Some variation between individuals can be noticed. The angiogram in (A) is the same image as in Figure 3(B) but in grayscale. The order of the subjects is the same as in Figure 5. Scale bars: 250 µm.

Figures 5 and 6 are OCT angiograms of the choriocapillaris from the eight normal subjects at the central fovea and at ∼6 mm temporal to the fovea. Both 3 mm×3 mm and 1.5 mm×1.5 mm field sizes were scanned, but only 1.5 mm×1.5 mm images are shown for better visualization. In some of the angiograms, sporadic, bright horizontal line artifacts can be seen, resulting from occasional rapid saccadic eye motion. Although there is some variation in the choriocapillaris pattern between individuals, the observed vascular structure agrees well with known architectural morphology, and OCT angiography appears to work in all eight subjects imaged. The observed variations in choriocapillaris structure in the OCT angiograms are likely caused by actual variation in structure between individuals and not the measurement artifacts. This emphasizes that care should be taken when comparing the choriocapillaris structure between different subjects.

No subject had severe astigmatism (no subject had more than 2.5D of refractive astigmatism), and it appears that mild to moderate levels of astigmatism do not severely affect the angiogram image quality. It should be noted, however, that severe astigmatism would likely reduce the transverse resolution and signal in OCT angiography. This problem could potentially be corrected by using corrective cylindrical lenses in the patient interface or by using adaptive optics. For imaging patients with poor fixation, hardware eye tracking or software motion correction may be necessary. Furthermore, in AMD patients with irregular RPE contours, a more sophisticated segmentation algorithm would be required to segment the choriocapillaris boundary accurately. It is also possible to make the spot size on the retina smaller by increasing the OCT beam diameter at the cornea, which may improve the transverse resolution of the *en face* OCT angiogram, but ocular aberrations will limit the effective resolution unless adaptive optics is used.

The major difference between this technique and fluorescein or ICG angiography is that OCT angiography is based on motion contrast. This has several advantages for clinical applications. Since OCT angiography does not require exogenous contrast agents, imaging measurements can be safely and frequently repeated. OCT angiography also images three-dimensional vascular structure and can image fine vascular structure in the choriocapillaris below the RPE. In addition, if the capillary erythrocyte speeds are too slow, OCT angiography would show lower speckle decorrelation values, which can be identified in the *en face* and cross-sectional OCT angiograms. This is an interesting property, since it suggests that OCT angiography may detect abnormalities in local perfusion before structural abnormalities such as vessel atrophy occur, which could be useful for early diagnosis. Unlike fluorescein or ICG angiography, OCT angiography does not detect dye leakage, which is beneficial for imaging the fenestrated choriocapillaris, but is limited in applications where alterations in vascular permeability are markers of disease.

The ultrahigh speed SSOCT prototype at the long 1060 nm wavelength has advantages over commercial spectral domain OCT (SDOCT) systems at 850 nm for choroidal imaging. Because of the normal curvature of the retina, OCT imaging away from the macula tends to result in a severe tilt of the retina. This in turn implies that it is difficult to avoid signal loss at all transverse locations, even with enhanced depth imaging (EDI) [33]. Compared with SDOCT, SSOCT has low sensitivity roll off with range and, combined with the deeper light penetration into tissue at longer wavelengths, it enables choroidal imaging with high signal-to-noise ratio (SNR) at all ranges. Good quality images can be achieved even with a severe retinal tilt (See the intensity image in Figure 1A as an example). Due to higher blood flow speed in the choroid than in the retina, SDOCT suffers from fringe washout artifacts that result in a significantly lower signal in blood vessels with fast blood flow speed. These artifacts are virtually absent in SSOCT [34]. Ultrahigh speed imaging with state-of-the-art SSOCT technology enables larger area coverage on the retina for a given total image acquisition time.

Because repeated B-scans are required for OCT angiography, the imaging area is smaller than *en face* intensity imaging at a given imaging speed. Next-generation commercial OCT systems are expected to have imaging speeds between 70 kHz and 100 kHz A-scan rates. At a 100 kHz A-scan rate, it should be possible to image 1.5 mm×1.5 mm fields with 400(horizontal)×200(vertical)×4(repeats) A-scans per volume, while maintaining the same transverse sampling density and total acquisition time as in this study, which imaged a 3 mm×3 mm field at 400 kHz with 800(horizontal)×400(vertical)×4(repeats) A-scans per volume. It should be also possible to extend the field size up to 2 mm by critical sampling, instead of oversampling by 1.33× as seen in the present study. Field sizes between 1.5 mm×1.5 mm and 2 mm×2 mm are relatively small, but it could still enable clinical studies with next-generation commercial OCT systems if the desired scan position is known. In general, further advances in OCT technology and imaging speed are expected to provide progressively larger retinal coverage in the future.

The current study was limited in that only relatively younger normal subjects were imaged. Normal subjects have better ocular stability and reduced ocular opacities when compared with typical retinal patients. Since only younger subjects were imaged, normal changes in the choriocapillaris associated with aging were not investigated. Further engineering is required to develop an ultrahigh speed SSOCT prototype for use in the ophthalmology clinic. Clinical imaging studies on patients with retinal disease as well as age-matched normal subjects are required in order to assess the ultimate clinical utility of OCT angiography of the choriocapillaris.

## Conclusions

The study demonstrated *in vivo* OCT angiography of the choriocapillaris in healthy volunteers by using long wavelength, ultrahigh speed SSOCT. Fine vascular structure could be imaged using motion contrast, without the need for exogenous contrast agents. OCT angiograms at different depths and fundus locations were consistent with known architectural morphology from early histological and electron micrograph corrosion casting findings. More extensive clinical imaging studies on patients with retinal disease and age-matched normal subjects are required in order to assess the ultimate utility of OCT angiographic imaging of the choriocapillaris. However, OCT and OCT angiography have the advantage in that they enable integrated imaging of both retinal and choroidal structure and vasculature. Since alterations in choriocapillaris structure are implicated in many retinal diseases, OCT angiography promises to enable cross-sectional as well as longitudinal studies in patients for a better understanding of pathogenesis, early diagnosis of diseases, treatment response monitoring, and improved efficiency of pharmaceutical development.

## Supporting Information

Figure S1 **Panoramic wide field of view OCT angiogram of the choriocapillaris spanning ∼32 mm on the retina.** The same images in Figure 2 are shown but with a higher pixel density to avoid cropping the wide field of view images. Scale bars: 1.5 mm.
(TIF)

Video S1 ***En face*** **OCT angiography and intensity flythrough in depth.** Top and bottom rows show *en face* OCT angiograms and intensity images, respectively, at the central fovea, ∼6 mm temporal, and ∼12 mm nasal to the fovea, from left to right. 1.5 mm×1.5 mm field of view for each fundus location.
(AVI)
