# Supplementary figures and images for: Choriocapillaris and Choroidal Microvasculature Imaging with Ultrahigh Speed OCT Angiography
Source: PLoS One. 2013 Dec 11;8(12):e81499. doi: 10.1371/journal.pone.0081499 (PMC3859478; doi:10.1371/journal.pone.0081499)

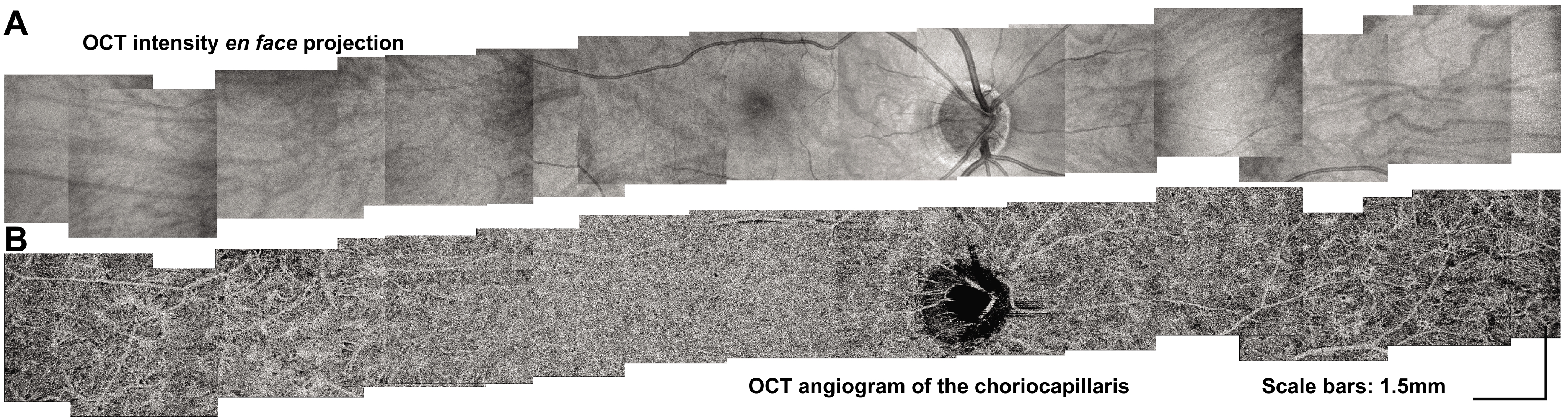

Supplement: Figure S1 — Panoramic wide field of view OCT angiogram of the choriocapillaris spanning ∼32 mm on the retina. The same images in Figure 2 are shown but with a higher pixel density to avoid cropping the wide field of view images. Scale bars: 1.5 mm. (TIF) [file pone.0081499.s001.tif]
